# Metaverse dependency as a psychosomatic risk of immersive platforms: development and validation of the Metaverse Dependency-Related Experiences Scale

**DOI:** 10.3389/fpsyg.2026.1814271

**Published:** 2026-09-23

**Authors:** Takuto Homma, Naoki Konishi, Ryohei Umetani, Jin Kato, Takemasa Yokoyama

**Affiliations:** 1 Department of Information Technology and Human Factors, National Institute of Advanced Industrial Science and Technology, Tsukuba, Japan; 2 Organization for the Strategic Coordination of Research and Intellectual Property, Meiji University, Suginami, Japan; 3 Faculty of Social Sciences, Hokuriku Gakuin University, Kanazawa, Japan

**Keywords:** dependence, metaverse, Metaverse Dependency-Related Experiences Scale (MDRES), scale development, social virtual reality

## Abstract

**Introduction:**

With the continuous expansion of socially immersive “metaverse” platforms, concerns regarding dependence-like tendencies associated with avatar-based virtual environments have grown. However, validated instruments that capture the psychosomatic and immersion-related characteristics central to such environments remain scarce. To address this gap, this study developed and preliminarily validated the Metaverse Dependency-Related Experiences Scale (MDRES), a self-report measure that assesses dependence-like tendencies in communication-oriented virtual environments.

**Methods:**

This research comprised two studies. Study 1 included the following two phases: Phase 1 involved qualitative item generation based on open-ended responses from active metaverse users, while Phase 2 involved item reduction and exploratory factor analysis (EFA) using the candidate items. Study 2 conducted confirmatory factor analysis and examined associations between the MDRES and theoretically relevant external variables.

**Results:**

In Study 1 Phase 1, qualitative responses were utilized to generate an initial pool of 52 candidate items. In Study 1 Phase 2, these 52 candidate items were administered, and EFA identified a six-factor, 39-item structure, including Reality Avoidance (α = 0.93), Confusion Between Physical and Virtual Spaces (α = 0.91), Awareness of Physical Symptoms (α = 0.85), Sleep Disturbance-Like Symptoms (α = 0.89), Physical Discomfort/Sensory Abnormalities (α = 0.85), and Stress Relief (α = 0.54). In Study 2, the six-factor structure received initial confirmatory support, while the MDRES subscales exhibited theoretically coherent associations with psychological distress, mood, somatic symptoms, mental health-related indicators, and game engagement.

**Discussion:**

Overall, the MDRES highlights the multidimensional, psychosomatic, and ambivalent nature (risk-related and stress-relieving) of metaverse involvement and offers a conceptually grounded tool for future work on dependence processes in immersive virtual environments.

## 1 Introduction

With the rapid advancement of virtual reality (VR) technology, the metaverse–shared virtual spaces on the Internet–has emerged as a significant technological and social development. The term “metaverse” originates from Neal Stephenson’s novel Snow Crash (Stephenson, 1992), where it combines “meta” and “universe” to describe an immersive, computer-generated environment. Contemporary examples include platforms such as VRChat, Rec Room, and Horizon Worlds that allow users to communicate and interact through avatars in user-created virtual worlds. For instance, VRChat is accessible through VR headsets, PCs, and mobile devices. Data from Steam (2026) show that the peak concurrent user count in January 2026 was 73,791. Given VRChat’s availability across multiple platforms, the actual user base is likely to be significantly larger. These developments illustrate how the metaverse enables social interactions among geographically distant individuals in immersive digital environments.

Beyond entertainment, the metaverse is being increasingly applied in fields such as education and business. According to PWC (2022) US Metaverse Survey, 50% of consumers described the metaverse as an exciting experience, 66% of business leaders reported that their organizations had already initiated metaverse-related projects, and 82% planned to incorporate it into business activities within the next 3 years. Demand Sage (2025) estimated that by 2026, there will be over 600 million monthly active users, nearly 40% of whom will be under 20 years of age, indicating strong adoption among younger generations.

Market forecasts suggest further expansion. Gartner (2022) predicted that by 2026, 25% of the global population would spend at least 1 h per day on the metaverse, and 30% of organizations would have metaverse-ready products or services. Similarly, Grand View Research Inc (2024) estimated the global market size at USD 105.4 billion in 2024, projecting a compound annual growth rate of 46.4% between 2025 and 2030. Other sources such as Coolest Gadgets (2025) forecast that the market could approach USD 93.6 billion by 2025 and USD 3 trillion by 2033. This rapid growth has been reinforced by corporate investments, most notably Facebook’s rebranding to Meta Platforms, Inc., in 2021, and by broader social factors such as the COVID-19 pandemic, which accelerated the shift toward online interaction and virtual engagement.

The rapid growth of metaverse users and the heightened sense of immersion may, alongside its convenience, increase the risk of addictive use. Previous findings on online gaming and virtual reality dependency suggest that excessive immersion may lead to serious psychological and behavioral problems (Kuss and Griffiths, 2012; He et al., 2025). These concerns have often been discussed within the framework of behavioral addiction, which, although only gambling disorder is officially recognized in the *DSM-5*, has been conceptually extended to other excessive behaviors such as gaming and Internet use.

According to Griffiths (2005) component model of addiction, addiction is characterized by core elements, including tolerance, withdrawal, and loss of control. Specifically, tolerance refers to the need to increase the frequency or duration of a behavior to achieve the same level of satisfaction, withdrawal denotes unpleasant feelings or irritability when the behavior is stopped, and loss of control describes continued engagement despite attempts at self-regulation. Building on this framework, Kuss and Griffiths (2012) argued that online gaming addiction shares many central features with other behavioral addictions. Considering that many current metaverse platforms deliver game-like formats, it is plausible that excessive immersion in such virtual environments may lead to psychological and social difficulties, although causal evidence and prevalence estimates remain limited.

Because the metaverse is centered on VR-enabled virtual spaces, the platform design has prioritized deepening the users’ sense of immersion. Empirical evidence indicates that advances in real-time 3D rendering have increased both immersion and in-world behaviors such as virtual consumption and social interactions (Zhang et al., 2025). Conceptually, five VR affordances (embodiment, interactivity, navigability, sense ability, and createability) explain how technical progress translates into a richer user experience (Dincelli and Yayla, 2022). However, the same mechanism of heightened engagement may increase dependence. Regression analyses showed that device usage time and the level of spatial perception predicted addiction scores, implicating both usage duration and degree of immersion as risk factors (He et al., 2025). Thus, the metaverse’s pursuit of immersion appeared to amplify engagement while simultaneously increasing the potential for dependence, which was comparable to, and may even exceed, that documented for conventional online gaming addiction.

At the same time, recent work on problematic technology use cautions against pathologizing all intense engagement. Kardefelt-Winther (2014) proposed the notion of compensatory use, whereby individuals turn to digital media to cope with offline stressors or unmet needs. From this perspective, metaverse use can be viewed as a continuum ranging from adaptive immersion, which provides enjoyment or psychosocial support, to maladaptive dependence, which is associated with significant impairment. Dependence is thus better understood as a spectrum, where both beneficial and harmful outcomes may coexist.

Research on problematic or addictive technology use extends beyond gaming to the Internet, smartphones, and social media. Representative measures include the Internet Addiction Test (Young, 1998), Compulsive Internet Use Scale (Meerkerk et al., 2009), Problematic Internet Use Questionnaire (Demetrovics et al., 2008), and Generalized Problematic Internet Use Scale 2 (Caplan, 2010). For mobile devices, scales such as the Smartphone Addiction Scale – Short Version (Kwon et al., 2013), the Mobile Phone Problem Use Scale (Bianchi and Phillips, 2005), and the Smartphone Addiction Proneness Scale (Kim et al., 2014) are frequently employed. Platform-specific instruments include the Bergen Social Media Addiction Scale (Andreassen et al., 2017) and the Social Media Disorder Scale (van Den Eijnden et al., 2016). Across these families, addiction is typically operationalized through the same behavioral components, namely, salience, tolerance, mood modification, withdrawal, relapse or loss of control, conflict, and negative consequences, reflecting the component model of behavioral addiction (Lemmens et al., 2009). In contrast, research on immersive technologies such as VR has focused largely on the quality of experience, including presence, immersion, and usability, rather than addiction (Witmer and Singer, 1998).

Nevertheless, emerging evidence links immersion to compulsive tendencies. Barreda-Ángeles and Hartmann (2022) adapted the Game Addiction Scale to VR application use and found that embodiment, more than presence, consistently predicted VR-related addictive tendencies. Their findings suggested that stronger sensory and bodily immersions may increase the likelihood of problematic or compulsive engagement. Madary and Metzinger (2016) identified addiction as a risk associated with the long-term use of immersive VR and highlighted the need to examine how the diagnostic criteria for VR addiction differ from those for Internet Use Disorder and related conditions. Given this observation, the use of the metaverse–featuring characteristics such as immersion, a sense of bodily presence, and interaction through avatars–may foster addictive tendencies that cannot be fully captured by existing frameworks for internet or gaming addiction alone. Furthermore, metaverse dependency is a relatively novel issue that may become more prominent in the future as the metaverse continues to expand. Therefore, developing a measurement scale tailored to metaverse dependency is crucial not only for assessing the extent of the problem after it has become severe but also for identifying risks at an early stage, before addictive tendencies become evident. Such a scale could help identify individuals at elevated risk of developing metaverse dependency and provide a foundation for designing preventive interventions and support methods.

However, validated instruments that specifically address dependence in socially immersive, metaverse-like environments remain scarce. Existing scales either focus on technology use in a general, device-specific, or platform-specific manner and rarely consider immersion-related mechanisms such as altered time perception, embodiment, or difficulty disengaging from virtual to physical contexts–processes central to metaverse use. Current measures of internet, gaming, smartphone, and social media addiction may not fully capture symptoms shaped by experiences unique to the metaverse, such as avatar embodiment, spatial presence, a sense of co-presence, and immersive social interactions. Specifically, the conceptual gap lies in the limited theoretical attention devoted to metaverse-specific dependency symptoms, whereas the empirical gap lies in the absence of a validated scale for measuring such symptoms. Hence, this research aims to address these gaps by developing a scale to assess dependency-related symptoms specific to metaverse use. The proposed metaverse-specific dependence scale was built on established behavioral addiction components. Additionally, it incorporates dimensions unique to immersive virtual social environments, including presence and embodiment, time distortion and prolonged engagement, and the continuity of social interaction and identity across virtual and physical realities. Situated at the intersection of technology addiction research and immersion psychology, this approach seeks to clarify how dependence may manifest in a metaverse context.

Furthermore, in line with the compensatory use framework (Kardefelt-Winther, 2014), the present scale is designed under the assumption that metaverse-related experiences span a spectrum that ranges from adaptive immersion to problematic dependence, rather than representing a single, homogeneous construct. As such, the scale is expected to capture multiple, partially overlapping dimensions that reflect both functional and dysfunctional aspects of metaverse use. This theoretical stance implies that the latent structure of metaverse dependence may be inherently multidimensional and only partially align with a strictly addictive factor, a point that is considered when interpreting the factor-analytic findings reported in later sections.

Previous studies have focused primarily on conventional gaming or general VR contexts, leaving the distinctive psychological and physical characteristics of the metaverse largely unexplored. Therefore, this study aimed to develop a scale to identify psychosomatic symptoms indicative of metaverse-specific dependence by examining metaverse users’ behaviors, attitudes, psychosomatic states, and communication patterns within virtual spaces. In doing so, we conceptualize metaverse use as a spectrum extending from highly immersive yet adaptive engagement to problematic dependence and seek to locate psychosomatic and behavioral symptoms along this continuum. The term metaverse is currently used across diverse contexts and platforms such as those centered on gaming (e.g., Fortnite, Roblox), social communication (e.g., VRChat, Cluster), and commerce (e.g., Amazon, BUY+). In particular, we focused on metaverse platforms that function as social networking services (SNS), where users form and maintain interpersonal relationships, because addictive use and psychosomatic complaints may be more likely to emerge in the context of continuous social interaction and feedback, especially when such interactions are experienced through avatars. Building on prior findings on SNS addiction, we expect similar social and psychological mechanisms to operate in avatar-based metaverse environments. Previous research has defined metaverse addiction as a compulsive and harmful dependence on immersive digital realms and interconnected experiences within virtual environments (Jia and Mvondo, 2025). However, metaverse dependency’s specific effects remain unclear. Although metaverse dependency may share certain characteristics with existing forms of behavioral dependency, such as gaming, social media, and internet dependency, it may also involve phenomena and effects unique to the metaverse. Therefore, this research conceptualizes metaverse dependency in terms of its overall effects in real-world contexts and aims to develop a scale for measuring metaverse dependency through an exploratory pilot study.

To guide the development and validation of the Metaverse Dependency-Related Experiences Scale (MDRES), this study addressed the following research questions (RQs). RQ1: What factor structure underlies metaverse dependence-related experiences? RQ2: Does the developed scale demonstrate an interpretable factor structure and sufficient internal consistency? RQ3: How are the MDRES subscales associated with theoretically relevant external variables, including psychological distress, game engagement, absorption, spatial presence, and other psychological or psychosomatic indicators?

The present research involved two studies. Study 1 comprised two phases. In Phase 1, “Qualitative Item Generation,” we administered an open-ended survey to collect free-text descriptions of virtual communication and metaverse-related experiences that could be linked to dependence-related tendencies. Based on these responses, candidate items for the MDRES were generated. In Phase 2, “Item Reduction and Exploratory Factor Analysis,” we administered a quantitative survey to metaverse users using the candidate items and performed exploratory factor analysis (EFA) to reduce the item pool and identify the initial factor structure of the MDRES. Study 2 examined this structure’s replicability and validity by conducting confirmatory factor analysis (CFA) and correlational analyses between the MDRES and theoretically relevant external variables. Figure 1 presents the overall flow of the present research, proceeding from Study 1 Phase 1 to Study 1 Phase 2 and then to Study 2.

**FIGURE 1:**
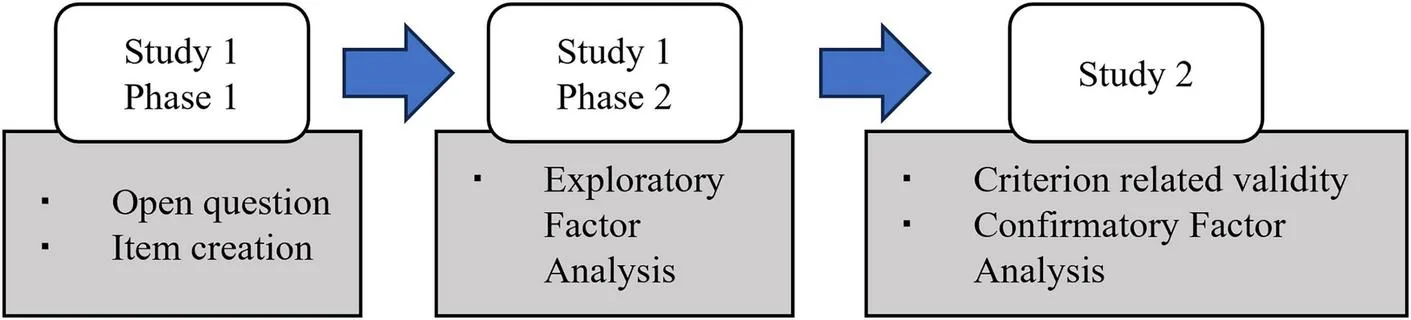
The flow of this research.

## 2 Study 1

### 2.1 Ethics approval

In accordance with local legislation (Act on the Protection of Personal Information), ethical review was not required as no personally identifiable information was collected. All procedures were conducted in compliance with relevant ethical guidelines and regulations. This study was subject to an internal ethical pre-screening by AIST’s Human Engineering Experimental Committee, which formally determined that the study did not fall under medical or life sciences research involving human subjects as defined by Japanese national guidelines (Reference Number: H2024-1471). The survey adhered to the principles outlined in the Act on the Protection of Personal Information and complied with JIS Q 15001 (Personal Information Protection Management System). All participants were informed that their participation was voluntary and that they had the right to withdraw at any time, with the assurance that their responses would be excluded from the dataset if they chose to withdraw.

### 2.2 Phase 1: qualitative item generation

A pilot study was conducted to explore and identify the behavioral, psychological, and physical experiences associated with the use of virtual environments and metaverse platforms. This preliminary investigation aimed to generate potential item contents to develop a comprehensive scale for assessing metaverse-related dependence. In Study 1, open-ended questions were used to capture a broad range of user experiences and symptoms before the formal item construction.

#### 2.2.1 Methods

We conducted an online survey via the survey company CrowdWorks Inc., and collected data from 50 individuals. All the respondents were registered users of CrowdWorks and were paid 300 yen (approximately USD 3). After excluding three individuals with suspicious data and unusual responses (e.g., reporting the use of a metaverse other than a social metaverse), we analyzed data for the remaining 47 participants (*M*_*age*_ = 39.40 ± 8.90, 29 males, 18 females).

All the survey questions were administered in Japanese. After providing informed consent, participants answered items assessing their sociodemographic characteristics and the influence of the behavioral characteristics of metaverse environments on their real-world experiences. For example, respondents were asked to provide free-text responses regarding their experiences in the metaverse, these experiences’ effects on their real lives, and metaverse use’s effects on their physical and mental health and values, thereby addressing both positive and negative aspects. Supplementary Table 1 presents the open-ended items employed in this study. Participants were asked to respond in an open-ended format to questions about their behavior in the metaverse, the impact of this behavior on their everyday lives, and their mental and physical symptoms and abnormalities.

#### 2.2.2 Results and discussion

Based on the open-ended responses derived from the preliminary survey, we obtained 611 free-text responses from 47 participants. The 611 responses were reviewed by three authors. Each free-text response was treated as the unit of analysis, and its content was summarized using a concise thematic label. After merging responses with substantively overlapping meanings, 238 distinct response units remained. The three authors then discussed the conceptual similarity of the remaining responses and organized them into thematic groups. Drawing on these qualitative responses and prior research (Kuss and Griffiths, 2012; Barreda-Ángeles and Hartmann, 2022; He et al., 2025), the authors drafted and refined the wording of candidate items, resulting in an initial 52-item pool covering addictive-tendency behaviors and symptoms, as well as metaverse-related immersion and reality confusion (Supplementary Table 2). Candidate items were generated and reviewed according to their relevance to metaverse-related experiences, clarity, specificity, and lack of redundancy. Disagreements regarding the classification of responses or item wording were resolved through discussion among the three authors. These items assessed behaviors in the metaverse, their impact on the real world, and the associated physical and psychological symptoms or abnormalities. They reflected psychosomatic symptoms and specific behaviors that may arise from engaging in metaverse content. In Study 1, the 52-item pool was administered to metaverse users to develop the MDRES.

### 2.3 Phase 2: item reduction and exploratory factor analysis

Phase 2 aimed to develop the MDRES, a measure of metaverse dependence, using the 52 items generated in the pilot study. To this end, we administered these items to individuals who actively used the metaverse and, based on these data, performed EFA to construct a reliable measure of metaverse dependence.

#### 2.3.1 Methods

##### 2.3.1.1 Participants

We conducted an online survey via the survey company CrowdWorks Inc., and collected data from 400 individuals. All the respondents were registered users of CrowdWorks and were paid 300 yen (approximately USD 3). After excluding 75 individuals with suspicious data and unusual responses (i.e., reporting the use of a metaverse other than a social metaverse, unclear usage duration, or unspecified content names), we analyzed data from the remaining 325 participants (*M*_*age*_ = 37.40 ± 8.38, 213 males, 112 females).

##### 2.3.1.2 Procedure

After obtaining informed consent, participants completed items assessing their sociodemographic characteristics and metaverse dependence. The MDRES candidate items address behaviors specific to metaverse environments and their influence on real-life experiences. All items and response options used in this study are presented in Supplementary Tables 3–5.

#### 2.3.2 Measurements

##### 2.3.2.1 Sociodemographic information

The sociodemographic questionnaire collected basic demographic variables–sex, age, prefecture of residence, marital status, and occupation–and details of metaverse use, namely the specific metaverse platform currently used and the length of use (in years).

##### 2.3.2.2 Metaverse dependency items

We used the 52 items created in the pilot study. Responses were collected using a 5-point Likert scale ranging from 1 (does not apply at all) to 5 (applies very much). Prior to responding, participants were presented with the following instructions: “the following items concern experiences and effects that may arise from engaging with content within virtual environments (metaverse). Please read each item carefully and select the option that best represents how accurately it applies to your current self.”

#### 2.3.3 Results

Among participants using VR, many primarily used VRChat services (see Supplementary Table 3). Descriptive statistics were calculated for each item. One item, “It became difficult to control my emotions,” exhibited a floor effect (*M* = 1.98, *SD* = 1.01) and was excluded from the EFA, as its mean minus one standard deviation fell below the minimum response option on the 5-point scale (M − *SD* < 1.00). This item was originally generated from open-ended responses wherein participants described the psychological or emotional changes that they experienced in relation to metaverse use. The basic statistics for each scale item are presented in Supplementary Table 5.

Next, EFA was conducted on the remaining 51 items. The number of factors was determined to be six, based on the Minimum Average Partial criterion. Assuming a six-factor solution, we performed EFA using the Minimum Residuals method with oblimin rotation. Items were iteratively screened using an absolute factor loading threshold of ≥0.40. Items that cross-loaded ≥0.40 on multiple factors or had communalities ≤0.16 were removed. Based on the resulting factor structure, we identified six distinct subscales, each representing a unique aspect of metaverse-related behaviors and experiences (Table 1). The subscales were as follows:

**TABLE 1:** Exploratory factor structure of the Metaverse Dependency-Related Experiences Scale: pattern loadings and communalities.

Panel A. Pattern matrix (primary loadings ≥ 0.40 in bold) and communalities								
**No.**	**Items (short)**	**F1**	**F2**	**F3**	**F4**	**F5**	**F6**	***h* ^2^**
26	Everything feels bothersome	**0.81**	0.03	0.01	0.02	0.00	−0.11	0.71
29	Weaker relationships	**0.78**	−0.05	0.04	0.05	0.08	0.10	0.70
27	Poor real-space communication	**0.75**	0.06	−0.10	0.13	−0.01	0.12	0.74
20	Less time with friends	**0.75**	0.04	−0.04	0.03	0.07	0.13	0.66
19	Socializing feels bothersome	**0.72**	−0.12	0.28	−0.14	0.04	0.04	0.54
39	Depressed more often	**0.66**	0.16	−0.04	0.16	−0.03	−0.16	0.73
50	Feeling down more often	**0.66**	0.06	0.04	0.07	−0.05	−0.08	0.53
46	Feeling lonely	**0.65**	0.09	−0.01	0.08	0.00	−0.05	0.56
36	Irritated more often	**0.62**	0.15	0.10	0.07	−0.03	−0.24	0.62
23	Less time with family	**0.60**	0.08	−0.14	0.09	0.10	0.22	0.58
30	More impatient	**0.47**	0.23	0.17	−0.06	0.07	−0.02	0.45
44	Same gestures (real–virtual)	−0.07	**0.85**	0.04	−0.05	0.01	−0.01	0.64
25	Use virtual terms in real	0.01	**0.71**	−0.07	−0.07	0.17	−0.08	0.54
31	Same communication style (real–virtual)	0.04	**0.70**	−0.06	0.06	0.11	0.17	0.67
45	Same facial expressions (real–virtual)	0.03	**0.69**	0.03	0.07	0.03	0.02	0.58
42	Virtual sounds heard in real	0.03	**0.65**	0.10	−0.04	−0.04	−0.08	0.42
41	Same locomotion (real–virtual)	0.00	**0.59**	−0.02	0.15	0.09	0.09	0.52
47	Virtual physics applied to real	0.32	**0.56**	0.08	0.04	−0.14	0.07	0.64
40	Same interpersonal distance (real–virtual)	0.25	**0.52**	−0.07	0.20	−0.02	0.08	0.64
51	Almost touch off-limits objects	0.12	**0.51**	0.03	0.13	0.06	0.01	0.47
2	Stiff shoulders	−0.02	0.04	**0.69**	−0.01	0.12	0.02	0.57
1	Eye strain	−0.07	−0.03	**0.68**	−0.11	0.19	0.01	0.58
4	Stiff neck	−0.09	−0.10	**0.57**	0.28	0.13	0.10	0.51
9	Head fatigue	−0.05	0.19	**0.55**	0.34	−0.01	−0.13	0.58
10	Slouching posture	0.24	0.16	**0.53**	0.02	−0.09	0.05	0.43
15	Decline in stamina	0.30	−0.05	**0.53**	0.07	0.13	0.12	0.58
11	Worsened eyesight	0.20	0.00	**0.51**	−0.03	0.11	−0.03	0.40
38	Dizzy on standing	0.23	0.14	−0.15	**0.63**	0.00	−0.01	0.71
18	Physical sluggishness	0.11	−0.07	0.30	**0.59**	0.14	−0.03	0.66
34	Headaches	0.14	0.05	0.09	**0.59**	0.11	−0.15	0.61
35	Ear pain	0.01	0.26	0.14	**0.53**	0.02	−0.05	0.56
33	Cybersickness	0.17	−0.01	0.00	**0.46**	0.11	0.00	0.35
5	Less sleep time	0.06	−0.02	−0.02	0.04	**0.86**	−0.04	0.74
3	Staying up late	0.00	0.07	0.04	−0.08	**0.82**	−0.02	0.70
8	Sleep-deprived	−0.08	0.07	0.08	0.09	**0.80**	0.05	0.77
22	Disordered daily routines	0.29	0.09	0.06	0.01	**0.45**	0.11	0.49
6	Less stress	−0.02	0.03	0.07	−0.18	0.04	**0.66**	0.50
37	Better sleep quality	0.01	0.13	0.01	0.27	−0.22	**0.47**	0.32
16	Elevated mood more often	0.08	0.28	0.10	−0.09	0.11	**0.42**	0.37
**Panel B. Factor intercorrelations**		**F1**	**F2**	**F3**	**F4**	**F5**	**F6**	
F1		–	0.55	0.22	0.53	0.23	0.09	
F2			–	0.09	0.46	0.28	0.11	
F3				–	0.21	0.49	0.01	
F4					–	0.18	−0.09	
F5						–	0.14	
F6							–	

Extraction: minimum residuals; Rotation: Oblimin. The number of factors was determined by the Minimum Average Partial criterion. Reported values are pattern coefficients; communalities (*h*^2^) are computed from reproduced correlations. Loadings < 0.40 are suppressed; primary loadings (| loading| ≥ 0.40) are boldfaced. Items with cross-loading ≥ 0.40 on multiple factors or with *h*^2^ ≤ 0.16 were excluded prior to the final solution. Rows for each factor are sorted in descending order of primary loading. Items are referenced by No.; short labels are shown here, and full item wordings are provided in Supplementary Table 1. Factors: F1 = Reality Avoidance; F2 = Confusion Between Real and Virtual Spaces; F3 = Awareness of Physical Symptoms; F4 = Sleep Disturbance-Like Symptoms; F5 = Physical Discomfort/Sensory Abnormalities; F6 = Stress Relief. Sample/fit: *N* = 325; KMO = 0.94; Bartlett’s test χ^2^ (*df* = 780) = 8383.79, *p* < 0.001. Coefficients rounded to two decimals.

(1) Reality Avoidance (reflecting avoidance of real-world communication and increased psychological burden),

(2) Confusion Between Physical and Virtual Spaces (representing the enactment in the real world of behaviors specific to virtual spaces),

(3) Awareness of Physical Symptoms (encompassing various bodily symptoms that may arise from metaverse engagement),

(4) Sleep Disturbance-Like Symptoms (capturing disturbances in sleep and daily routines related to metaverse use),

(5) Physical Discomfort/Sensory Abnormalities (describing discomfort and sensory irregularities resulting from physical strain in virtual environments), and

(6) Stress Relief (indicating positive effects such as stress reduction, derived from metaverse use).

Cronbach’s alpha was used to assess the reliability of the subscales. The values obtained were as follows: Reality Avoidance (α = 0.93), Confusion Between Physical and Virtual Spaces (α = 0.91), Awareness of Physical Symptoms (α = 0.85), Sleep Disturbance-Like Symptoms (α = 0.89), Physical Discomfort/Sensory Abnormalities (α = 0.85), and Stress Relief (α = 0.54).

Discriminant validity was also examined using the heterotrait–monotrait (HTMT) ratio of correlations. All 15 pairwise HTMT values ranged from 0.31 to 0.77 and were below the conservative threshold of 0.85, supporting the discriminant validity of the six subscales (Henseler et al., 2015; see Supplementary Table 6).

#### 2.3.4 Discussion

In Study 1 Phase 2, the EFA identified six subscales to measure behaviors and the psychological and physical impacts related to metaverse dependence. These subscales provide key indicators of how metaverse users’ experiences and behaviors are associated with their everyday lives in the physical world. Specifically, six subscales emerged: Reality Avoidance, Confusion Between Physical and Virtual Spaces, Awareness of Physical Symptoms, Sleep Disturbance-Like Symptoms, Physical Discomfort/Sensory Abnormalities, and Stress Relief (Supplementary Indicator). The findings underscore that the MDRES is not a unidimensional scale that measures a single level of dependence, but rather a multidimensional measure that captures the multifaceted experiences associated with metaverse dependence. Experiences linked to metaverse dependency may encompass multiple domains, including behavior, thoughts, and physical reactions. Therefore, the MDRES should be understood as a scale for assessing the multidimensional profile of risk and adaptation associated with metaverse use. Consistent with this multidimensional interpretation, all HTMT values were below the conservative threshold of 0.85 (Henseler et al., 2015), supporting the discriminant validity of the six MDRES subscales in Study 1. The Reality Avoidance subscale captures metaverse-specific content that may reflect negative experiences associated with problematic or dependence-related metaverse use, potentially reflecting the use of metaverse environments as a means of escaping from or coping with difficulties encountered in the physical world. In gaming addiction, excessive immersion has been shown to manifest as psychological and social problems such as depression, anxiety, social withdrawal, and interpersonal conflict (Lemmens et al., 2011; Pontes et al., 2015; Loton et al., 2016), and similar associations may also be observed in metaverse-related dependence. Conceptually, this factor may correspond to the conflict/problems component of the Game Addiction Scale (Lemmens et al., 2009).

The Confusion Between Physical and Virtual Spaces subscale may be interpreted in relation to the literature on Game Transfer Phenomena (GTP), which suggests that experiences within virtual or game environments carry over into real-world contexts through changes in visual and auditory perception, automatic thoughts, automatic behaviors, and involuntary actions (Ortiz de Gortari et al., 2011, 2015). Furthermore, the Multiple Dimensions of GTP (GTP-MDS) framework conceptualizes these experiences as multidimensional phenomena involving intrusive cognitions, perceptual distortions, hallucination-like experiences, and dissociation (Ortiz de Gortari and Diseth, 2022). From this perspective, the present factor may partially overlap with GTP-related concepts, as it reflects experiences wherein users report difficulties distinguishing between physical and virtual contexts and applying virtual experiences to real-world situations. Simultaneously, this factor should not be equated with GTP itself. Whereas GTP research has primarily focused on the involuntary transfer of gaming experiences into daily life, the MDRES assesses metaverse dependency–defined as metaverse use’s effects as they appear in real-world contexts.

The Awareness of Physical Symptoms, Sleep Disturbance-Like Symptoms, and Physical Discomfort/Sensory Abnormalities subscales aligned with adverse mental and physical effects previously reported in the literature on gaming, Internet, and SNS dependence. Gaming disorders have been associated with both depressive symptoms and major depression (Ostinelli et al., 2021). Reportedly, prolonged gaming negatively affects the musculoskeletal system (Tholl et al., 2022). Internet addiction has also been linked to impaired sleep quality and duration (Alimoradi et al., 2019; Kokka et al., 2021). Among those with SNS addiction, meta-analyses have reported adverse outcomes including depression, anxiety, poor health status, and sleep problems (Shannon et al., 2022; Ahmed et al., 2024; Chen et al., 2024). Because the present scale targeted dependence tendencies related to communication-oriented SNS-like metaverse use, these factors aligned well with adverse effects.

The Physical Discomfort/Sensory Abnormalities subscale may capture experiences that partially overlap with cybersickness and other forms of VR-induced discomfort. Cybersickness-related symptoms are generally regarded as acute adverse reactions that occur during or shortly after VR exposure. Prior research has identified VR motion sickness and cybersickness as key negative factors within social VR and broader virtual environments (Rebenitsch and Owen, 2016; Gong, 2021). However, the MDRES assesses metaverse dependency’s medium- to long-term effects as they appear within real-world contexts–including psychosomatic symptoms and dependence-related experiences associated with repeated or prolonged metaverse use–rather than only acute discomfort resulting from VR exposure. Therefore, this subscale may be better understood as capturing metaverse-related physical discomfort that may overlap with, but is not limited to, acute VR-induced symptoms.

The Stress Relief subscale warrants a different interpretation from the other dependency-related symptom factors. This subscale’s items reflect metaverse use’s positive aspects and users’ subjective perception that metaverse use provides relief from stress and negative emotions. For example, social VR has been associated with diminished feelings of loneliness and social anxiety, suggesting that it may alleviate these issues (Kenyon et al., 2023). Social activities such as interacting with friends may enhance feelings of connection and enjoyment, while play and creative activities may foster self-expansion (Barreda-Ángeles and Hartmann, 2022). Accordingly, the Stress Relief subscale is conceptually distinct from and should be interpreted separately from the risk-related dimensions of the MDRES. It should be treated as a Supplementary Indicator of the perceived positive effects of metaverse use rather than as a core indicator of dependency risk. As the Stress Relief subscale comprises only three items, the Cronbach’s alpha coefficient was relatively low. Therefore, we computed the average inter-item correlation among the three items, which was 0.28. This value fell within the recommended range of 0.15–0.50 for evaluating item homogeneity in brief scales (Briggs and Cheek, 1986; Clark and Watson, 1995). However, this result does not eliminate the limited reliability of the subscale, and caution is warranted when interpreting this subscale, as it remains unclear whether the current three items specifically and adequately measure stress relief.

## 3 Study 2

In Study 1, we developed the MDRES by collecting and refining items related to metaverse engagement and conducting an EFA. Study 2 aimed to provide an initial confirmatory test of the six-factor structure identified in Study 1 and examine the associations between the MDRES and theoretically relevant external variables. Specifically, we analyzed the associations between each MDRES subscale and several external variables–including psychological distress, mood, physical symptoms, and mental health-related indicators–to determine whether the MDRES would demonstrate theoretically consistent patterns of association with established psychological and health-related measures.

### 3.1 Methods

#### 3.1.1 Participants

We conducted an online survey via the survey company MSS, Inc., and collected data from 300 individuals. Participants were registered users of MSS, Inc., and were compensated for their participation with points distributed by the company. After excluding 40 individuals with suspicious data and unusual responses (i.e., responses indicating the use of a metaverse other than a social metaverse, or responses where the name of the content was unspecified), we analyzed data for the remaining 260 participants (*M*_*age*_ = 36.85, *SD* = 9.92, 164 males, 96 females). In Study 2, participants also reported the devices they primarily used to access metaverse platforms. Many participants used PC-connected or standalone VR goggles, followed by PCs, desktops, and smartphones (see Supplementary Table 7 for details of the devices used).

#### 3.1.2 Procedure

After providing informed consent, participants completed items assessing their sociodemographic characteristics (the same as those used in Study 1), the MDRES, and the scales used to examine associations with theoretically relevant external variables. All the items and response options used in this study are presented in Supplementary Tables 5, 7. For the MDRES, we used 39 items extracted through the EFA conducted in Study 1. Responses were collected using a 5-point Likert scale ranging from 1 (does not apply at all) to 5 (applies very much). The instructions for the MDRES were the same as those used in Study 1.

#### 3.1.3 Measurements

The following scales were used to assess psychological and health-related variables in this study.

##### 3.1.3.1 Somatic Symptom Scale-8

The linguistically validated Japanese version of the Somatic Symptom Scale-8 (SSS-8) was used to assess somatic symptom sensitivity. The scale consists of eight items, with responses collected using a 5-point Likert scale ranging from 1 (not at all bothered) to 5 (very bothered). In this study, the SSS-8 total score and item-level scores, including symptoms such as dizziness and sleep problems, were employed to examine associations with the MDRES subscales. Prior to responding, participants were presented with the following instruction: “During the past week, to what extent have you been bothered by the following physical symptoms?”

##### 3.1.3.2 Kessler Psychological Distress Scale

The Kessler Psychological Distress Scale was used to assess psychological distress. Responses were collected using a 5-point Likert scale ranging from 1 (never) to 5 (always). In this study, the total score was utilized as an indicator of psychological distress. Prior to responding, participants were presented with the following instruction: “During the past 30 days, how often did the following occur?”

##### 3.1.3.3 Brief Mood Checklist–Japanese version

A linguistically validated Japanese version of the Brief Mood Checklist was used to assess the mood. The scale consists of nine items, with responses collected using a 7-point Likert scale ranging from 0 (does not apply at all) to 6 (applies very much). In this study, positive and negative affect scores were utilized to examine associations between mood states and the MDRES subscales. Prior to responding, participants were presented with the following instructions: “Please consider how much each of the following statements applies to you at present and select the most appropriate option.”

##### 3.1.3.4 Game Engagement Questionnaire–Japanese version

A linguistically validated Japanese version of the Game Engagement Questionnaire (GEQ-J) was used to assess game engagement. The scale consists of 15 items, with responses collected using a 5-point Likert scale ranging from 1 (does not apply) to 5 (applies). In this study, the GEQ-J subscales of assimilation, absorption, flow, and spatial presence were utilized as indicators of immersive and virtual-environment-related engagement. Prior to responding, participants were presented with the following instruction: “Please read the following statements and select the option that best applies to you.”

##### 3.1.3.5 University Personality Inventory

The linguistically validated Japanese version of the University Personality Inventory (UPI) was employed to assess mental health status and psychological symptoms. The scale consists of 60 items, with responses collected using a dichotomous scale (○/×), coded as 1 = ○ (Yes) and 0 = × (No). In this study, the following UPI subscales were employed: hypochondriacal symptoms, depressive tendency, autonomic symptoms, interpersonal anxiety/inferiority, obsessive–compulsive tendency, persecutory/ideas-of-reference ideation, and positive items. Prior to responding, participants were presented with the following instruction: “The questions below list things that many people often experience. We asked them to help us understand and promote your health. Please read them carefully, in numerical order. For each item, if you have sometimes felt or experienced it during the past year, mark a circle (○) next to its number; if you have not, mark a cross (×) (choose one response per item). Your responses concern your personal matters and will never be disclosed to others or used for any purpose other than the one stated above, so please answer honestly.”

### 3.2 Data analysis

In Study 2, we conducted a CFA of the 39-item MDRES to examine whether the six-factor structure identified in Study 1 would be supported by an independent sample. Six latent factors were allowed to correlate, and each item was specified to load only its respective factors. Cross-loading or correlated residuals were not allowed. The model parameters were estimated using maximum likelihood. Next, we computed the Pearson product–moment correlations and partial correlations between the MDRES subscales and external measures to examine whether the MDRES subscales exhibited theoretically meaningful associations with relevant psychological and health-related variables.

### 3.3 Results

#### 3.3.1 Factor structure of the MDRES

Before examining associations with theoretically relevant external variables, we conducted a CFA to test the six-factor structure of the 39-item MDRES identified in Study 1. In this model, all items were specified to load onto their respective latent factors (Reality Avoidance, Confusion Between Physical and Virtual Spaces, Awareness of Physical Symptoms, Sleep Disturbance-Like Symptoms, Physical Discomfort/Sensory Abnormalities, and Stress Relief) with no cross-loadings or correlated residuals. The initial model did not demonstrate adequate overall fit, χ^2^(687) = 1940.17, *p* < 0.001, χ^2^/df = 2.82, comparative fit index (CFI) = 0.84, Tucker–Lewis index (TLI) = 0.83, root mean square error of approximation (RMSEA) = 0.08, 90% confidence interval (CI) [0.07, 0.08], goodness of fit index (GFI) = 0.76. Although the standardized factor loadings were in the expected direction and were generally moderate to high, the overall fit indices did not provide sufficient support for the initially specified simple-structure model (see Supplementary Table 8 for item-level standardized factor loadings and Supplementary Table 9 for latent factor correlations).

Because the initial model did not demonstrate adequate fit, we examined whether certain items might be associated with their primary as well as secondary factors. Starting from the initially specified six-factor model, modification indices were inspected only for omitted paths from latent factors to items assigned primarily to other factors. Beginning with the path showing the largest modification index, cross-loadings were estimated sequentially and freely, and the model was re-estimated after each modification. No correlated residuals were added. The modification process was stopped after 32 cross-loadings had been added, at which point the CFI and TLI exceeded 0.90 and the RMSEA was approximately 0.05. The modified model demonstrated substantially improved and generally acceptable fit, χ^2^(655) = 1202.29, *p* < 0.001, χ^2^/*df* = 1.84, CFI = 0.93, TLI = 0.92, RMSEA = 0.05, 90% CI [0.05, 0.06], and GFI = 0.84 (see Supplementary Table 10 for the item-level standardized factor loadings and Supplementary Table 11 for the latent factor correlations).

#### 3.3.2 Associations with theoretically relevant external variables

Next, to evaluate the criterion-related validity of the MDRES, Pearson’s partial correlations (partial *r*) were computed between the MDRES subscales and various external measures. Because the MDRES subscales were strongly intercorrelated, this approach was used to estimate the unique association between each MDRES subscale and the external measures. To account for multiple testing, the Benjamini–Hochberg (BH) false discovery rate correction (Benjamini and Hochberg, 1995) was applied separately to the family of 15 correlations examined for each MDRES subscale (Table 2).

**TABLE 2:** Partial correlations between the Metaverse Dependency-Related Experiences Scale subscales and external measures (controlling for the remaining subscales).

Measure	F1 avoidance	F2 confusion	F3 physical	F4 sleep-like	F5 discomfort	F6 stress relief
SSS-8	0.08 (0.304, 0.465)	0.00 (0.952, 0.976)	0.12 (0.117, 0.357)	0.11 (0.126, 0.309)	0.08 (0.309, 0.908)	0.08 (0.258, 0.484)
Kessler Psychological Distress Scale (K10)	**0.20 (0.005, 0.025)**	0.08 (0.277, 0.806)	0.04 (0.628, 0.628)	−0.04 (0.575, 0.616)	0.02 (0.784, 0.917)	−0.03 (0.707, 0.758)
Negative affect (BMC-J)	**0.21 (0.004, 0.025)**	0.03 (0.723, 0.976)	0.08 (0.281, 0.534)	0.01 (0.852, 0.852)	−0.02 (0.795, 0.917)	0.00 (0.950, 0.950)
Positive affect (BMC-J)	−0.06 (0.391, 0.533)	0.02 (0.753, 0.976)	−0.04 (0.566, 0.628)	−0.05 (0.489, 0.598)	−0.04 (0.599, 0.917)	**0.30 (**<**0.001, 0.010)**
Assimilation (GEQ-J)	0.08 (0.258, 0.465)	**0.28 (<0.001, 0.008)**	−0.06 (0.416, 0.624)	0.07 (0.333, 0.500)	0.05 (0.456, 0.908)	0.13 (0.078, 0.225)
Absorption (GEQ-J)	**−0.21 (0.004, 0.025)**	**0.26 (<0.001, 0.008)**	0.05 (0.528, 0.628)	**0.30 (<0.001, 0.015)**	−0.07 (0.349, 0.908)	**0.23 (0.002, 0.010)**
Flow (GEQ-J)	0.11 (0.148, 0.370)	**0.21 (0.004, 0.015)**	−0.08 (0.285, 0.534)	0.17 (0.022, 0.165)	−0.02 (0.781, 0.917)	0.04 (0.625, 0.758)
Spatial Presence (GEQ-J)	**−0.16 (0.027, 0.101)**	**0.21 (0.004, 0.015)**	0.05 (0.477, 0.628)	0.11 (0.144, 0.309)	−0.05 (0.484, 0.908)	**0.23 (0.002, 0.010)**
Hypochondriacal symptoms (UPI)	−0.13 (0.068, 0.204)	0.07 (0.376, 0.806)	0.20 (0.007, 0.105)	0.05 (0.477, 0.598)	0.07 (0.330, 0.908)	−0.04 (0.567, 0.758)
Depressive tendency (UPI)	0.03 (0.721, 0.832)	0.01 (0.900, 0.976)	0.11 (0.119, 0.357)	0.10 (0.165, 0.309)	0.00 (0.955, 0.959)	−0.15 (0.037, 0.139)
Autonomic symptoms (UPI)	−0.06 (0.442, 0.553)	0.07 (0.375, 0.806)	0.08 (0.251, 0.534)	0.05 (0.518, 0.598)	0.10 (0.194, 0.908)	−0.03 (0.703, 0.758)
Interpersonal anxiety/inferiority (UPI)	0.00 (0.949, 0.964)	0.01 (0.911, 0.976)	0.12 (0.093, 0.357)	0.14 (0.061, 0.228)	0.00 (0.959, 0.959)	−0.12 (0.105, 0.225)
Obsessive–compulsive tendency (UPI)	−0.09 (0.202, 0.433)	0.05 (0.467, 0.876)	**0.15 (0.042, 0.315)**	0.13 (0.073, 0.228)	0.05 (0.480, 0.908)	−0.05 (0.483, 0.725)
Persecutory/ideas-of-reference ideation (UPI)	0.07 (0.310, 0.465)	0.00 (0.976, 0.976)	0.07 (0.329, 0.548)	0.13 (0.076, 0.228)	−0.04 (0.615, 0.917)	−0.07 (0.334, 0.557)
Positive items (UPI)	0.00 (0.964, 0.964)	−0.02 (0.764, 0.976)	0.04 (0.605, 0.628)	−0.07 (0.316, 0.500)	−0.07 (0.372, 0.908)	0.12 (0.096, 0.225)

Entries are *r* (unadjusted *p*, BH-adjusted *p*): the correlation coefficient *r* with the two-tailed *p*-value in parentheses. BH-adjusted *p*-values <0.05 are shown in bold.

#### 3.3.3 MDRES subscale: Reality Avoidance

Reality Avoidance showed a positive correlation with the Kessler Psychological Distress Scale (*r* = 0.20, unadjusted *p* = 0.005, BH-adjusted *p* = 0.025), indicating that higher reality avoidance was associated with greater psychological distress. Additionally, it was positively correlated with negative affect on the Brief Mood Checklist (*r* = 0.21, unadjusted *p* = 0.004, BH-adjusted *p* = 0.025). However, reality avoidance showed negative correlations with absorption on the GEQ-J (*r* = −0.21, unadjusted *p* = 0.004, BH-adjusted *p* = 0.025).

#### 3.3.4 MDRES subscale: confusion between physical and virtual spaces

Confusion between physical and virtual spaces was positively associated with several dimensions of game engagement in the GEQ-J, including assimilation (*r* = 0.28, unadjusted *p* < 0.001, BH-adjusted *p* = 0.008), absorption (*r* = 0.26, unadjusted *p* < 0.001, BH-adjusted *p* = 0.008), flow (*r* = 0.21, unadjusted *p* = 0.004, BH-adjusted *p* = 0.015), and spatial presence (*r* = 0.21, unadjusted *p* = 0.004, BH-adjusted *p* = 0.015). This suggests that individuals who experience confusion between physical and virtual spaces tend to report greater engagement with virtual environments.

#### 3.3.5 MDRES subscale: Sleep Disturbance-Like Symptoms

Sleep Disturbance-Like Symptoms were positively correlated with absorption (*r* = 0.30, unadjusted *p* < 0.001, BH-adjusted *p* = 0.015) on the GEQ-J, suggesting that sleep disturbances are linked to higher levels of engagement and absorption in virtual environments.

#### 3.3.6 MDRES subscale: Stress relief (Supplementary Indicator)

Stress relief was positively correlated with positive affect (*r* = 0.30, unadjusted *p* < 0.001, BH-adjusted *p* = 0.010), absorption (*r* = 0.23, unadjusted *p* = 0.002, BH-adjusted *p* = 0.010), and spatial presence (*r* = 0.23, unadjusted *p* = 0.002, BH-adjusted *p* = 0.010) on the GEQ-J, suggesting that individuals who experience greater stress relief tend to report higher positive affect.

#### 3.3.7 MDRES subscale: Awareness of Physical Symptoms (correlation with SSS-8 items)

Awareness of Physical Symptoms did not correlate with the other composite scales; therefore, partial correlations were computed between this subscale and the relevant items from the SSS-8, which captures autonomic-like symptoms (Table 3). A significant correlation was found between Awareness of Physical Symptoms and the SSS-8 item “Trouble sleeping” (*r* = 0.20, unadjusted *p* = 0.006, BH-adjusted *p* = 0.040), “Feeling tired or having low energy” (*r* = 0.19, unadjusted *p* = 0.010, BH-adjusted *p* = 0.040), and “Headache” (*r* = 0.18, unadjusted *p* = 0.017, BH-adjusted *p* = 0.045), suggesting that these symptoms were linked to sleep disturbance and somatic complaints.

**TABLE 3:** Item-level partial correlations with the Autonomic Symptoms subscale (SSS-8).

SSS-8 items	F3 physical	F4 sleep-like	F5 discomfort
Headache	**0.18 (0.017, 0.045)**	0.06 (0.442, 0.523)	0.12 (0.114, 0.405)
Feeling tired or having low energy	**0.19 (0.010, 0.040)**	0.13 (0.087, 0.232)	−0.11 (0.152, 0.405)
Pain in the arms, legs, or joints	0.06 (0.416, 0.555)	0.06 (0.387, 0.523)	0.04 (0.614, 0.814)
**Dizziness**	−0.12 (0.112, 0.179)	0.15 (0.044, 0.176)	**0.28 (<0.001, 0.008)**
Gastrointestinal problems	0.00 (0.959, 0.959)	0.05 (0.458, 0.523)	0.08 (0.281, 0.562)
Back or low-back pain	0.15 (0.045, 0.090)	0.07 (0.346, 0.523)	0.03 (0.683, 0.814)
Chest pain or shortness of breath	0.05 (0.515, 0.588)	−0.03 (0.734, 0.734)	0.01 (0.888, 0.888)
**Trouble sleeping**	**0.20 (0.006, 0.040)**	**0.20 (0.005, 0.040)**	0.03 (0.712, 0.814)

Entries are *r* (unadjusted *p*, BH-adjusted *p*): the correlation coefficient *r* with the two-tailed *p*-value in parentheses. BH-adjusted *p*-values <0.05 are shown in bold.

#### 3.3.8 MDRES subscale: Sleep Disturbance-Like Symptoms (correlation with SSS-8 items)

Sleep Disturbance-Like Symptoms did not correlate with the other composite scales; therefore, partial correlations were computed between this subscale and the relevant items from the SSS-8, which captures autonomic-like symptoms (Table 3). A significant correlation was found between Sleep Disturbance-Like Symptoms and the SSS-8 item “Trouble sleeping” (*r* = 0.20, unadjusted *p* = 0.005, BH-adjusted *p* = 0.040), suggesting that these symptoms were linked to sleep disturbance (Table 3).

#### 3.3.9 MDRES subscale: Physical Discomfort/Sensory Abnormalities (correlation with SSS-8 items)

Physical Discomfort and Sensory Abnormalities did not correlate with the other composite scales; therefore, partial correlations were computed between this subscale and the relevant items from the SSS-8, which captures autonomic-like symptoms (Table 3). A significant correlation was found between Physical Discomfort/Sensory Abnormalities and the SSS-8 item “Dizziness” (*r* = 0.28, unadjusted *p* < 0.001, BH-adjusted *p* = 0.008).

### 3.4 Discussion

Study 2 examined the factorial structure of the MDRES developed in Study 1 and its associations with theoretically relevant external variables. The initially specified six-factor model identified in the exploratory analyses did not demonstrate adequate overall fit in the CFA. After 32 cross-loadings were freely estimated based on the modification indices, the modified model demonstrated substantially improved and generally adequate fit. However, because these modifications were derived from the same CFA sample, the modified structure should be interpreted as providing preliminary rather than independent confirmatory evidence. We then analyzed the associations between each MDRES subscale and external variables, including psychological distress, mood, physical symptoms, mental health-related indicators, and game engagement characteristics. These variables were selected because they were theoretically expected to relate to specific aspects of metaverse dependence-related experiences.

Psychological distress and negative mood were expected to be associated with avoidance-oriented use. Further, game engagement, absorption, flow, and spatial presence were expected to relate to immersive and virtual-environment-related experiences. Finally, somatic symptoms and UPI-based mental health indicators were expected to relate to physical symptom awareness and psychosomatic complaints.

The Reality Avoidance subscale showed positive associations with indicators of psychological distress, including the Kessler Psychological Distress Scale and negative affect. This was likely because several items explicitly reflected the avoidance or coping motives linked to negative emotions. This finding is consistent with prior studies suggesting that escape-oriented internet or gaming use may be associated with distress and coping deficits (Demetrovics et al., 2011; Kardefelt-Winther, 2014; Melodia et al., 2022). In contrast, reality avoidance was negatively correlated with the GEQ-J dimensions of absorption, suggesting that avoidance tendencies may be associated with lower immersive engagement, possibly through rumination-related attentional difficulties (Whitmer and Gotlib, 2013) or reduced attentional allocation under distress (Wirth et al., 2007). These results suggest that avoidance-oriented motives may co-occur with psychological strain, indicating a theoretically interpretable pattern of association.

The Confusion Between Physical and Virtual Spaces subscale showed small to moderate positive correlations with the GEQ-J subscales of assimilation, absorption, flow, and spatial presence. The Confusion Between Physical and Virtual Spaces subscale is linked to immersive engagement, presence, and virtual-environment-related experiences. This interpretation aligns with presence theory, which holds that immersive VR produces a sense of “being there” and makes users respond realistically to virtual events (Slater, 2009). Moreover, it aligns with research on embodiment and body ownership, highlighting that virtual or visually mediated bodies alter self-location and body ownership (Petkova and Ehrsson, 2008).

In the SSS-8 item-level analyses, both the Awareness of Physical Symptoms and Physical Discomfort/Sensory Abnormalities subscales showed significant positive partial correlations with specific SSS-8 items. The Awareness of Physical Symptoms subscale was positively associated with three SSS-8 items: “Feeling tired or having low energy, “Headache,” and “Trouble sleeping.” Given that previous research has documented associations between somatic symptoms and sleep disturbances (Ionescu et al., 2021), these findings represent a theoretically interpretable pattern of associations. The Physical Discomfort/Sensory Abnormalities subscale was positively associated with the SSS-8 “Dizziness” item. Because this subscale encompasses dizziness-like symptoms associated with metaverse use, its association with dizziness is theoretically consistent with the content of the subscale.

Positive correlations with absorption were observed in the Sleep Disturbance-Like Symptoms subscale. Previous studies have indicated that problematic or extended nighttime gaming may delay sleep onset and impair sleep quality (Higuchi et al., 2005; Weaver et al., 2010). Although no overall correlation with the SSS-8 total score emerged, item-level analyses revealed associations with “Sleep problems,” conceptually aligned with sleep disturbances (van Leeuwen et al., 2024). These results provide additional evidence of theoretical coherence in the associations between the MDRES subscales and related external variables.

The stress relief factor correlated positively with positive affect, GEQ-J absorption, and spatial presence. Given that the item content explicitly involved enjoyment and stress reduction, these relationships were theoretically expected and are consistent with prior findings that metaverse and gaming experiences may be associated with mood recovery and relaxation (Barreda-Ángeles and Hartmann, 2022; Kenyon et al., 2023).

## 4 General discussion

### 4.1 Summary of the present findings

This study developed the MDRES, a self-report measure of metaverse-related dependence, and provided preliminary evidence of its psychometric soundness and conceptual utility in socially immersive contexts. In this study, the term “metaverse-related dependence” referred to dependence-like tendencies within a behavioral addiction framework rather than to a formal clinical diagnosis of “addiction” in the *DSM-5* sense. Across a pilot study and two surveys, the MDRES provided preliminary evidence for a coherent six-factor structure and acceptable internal consistency for most subscales. The factor structure was identified through EFA in Study 1 and provisionally supported by CFA in Study 2, though the CFA fit indices indicated that further model refinement is needed. The following six domains emerged: Reality Avoidance, Confusion Between Physical and Virtual Spaces, Awareness of Physical Symptoms, Sleep Disturbance-Like Symptoms, Physical Discomfort/Sensory Abnormalities, and Stress Relief (Supplementary Indicator). However, these subscales should not be interpreted as representing symptoms of a single clinical addiction. Of the six subscales, Reality Avoidance most directly reflects an addiction-related process, insofar as metaverse use may serve as a means of escaping from or avoiding difficulties in the real world. Confusion Between Physical and Virtual Spaces primarily captures cognitive and perceptual experiences associated with immersive use. In contrast, Awareness of Physical Symptoms, Sleep Disturbance-Like Symptoms, and Physical Discomfort/Sensory Abnormalities assess accompanying physical and sleep-related experiences. Stress Relief differs from these risk-related subscales in that it reflects a perceived benefit of metaverse use. Accordingly, the MDRES should not be regarded as a diagnostic measure of metaverse addiction, but rather as a preliminary multidimensional scale for assessing metaverse-related dependence-like tendencies and the associated psychological and physical experiences.

These domains suggested that immersive virtual experiences comprise both maladaptive and adaptive facets, spanning affective, cognitive, and somatic processes. Internally, an interpretable six-factor structure was supported by both exploratory and confirmatory factor analyses, although the confirmatory fit indices were acceptable rather than optimal. Externally, relationships with theoretically relevant external variables such as psychological distress, immersion-related experiences, health anxiety-like bodily awareness, sleep-related complaints, and dizziness as a marker of cybersickness were broadly consistent with theoretical expectations. Several associations remained significant even after controlling for inter-correlations among subscales, suggesting that each domain, while correlated with others, retained non-redundant variance.

Additionally, these findings also address the three RQs posed in the Introduction. Regarding RQ1, the EFA identified six factors underlying metaverse dependence-related experiences–namely, Reality Avoidance, Confusion Between Physical and Virtual Spaces, Awareness of Physical Symptoms, Sleep Disturbance-Like Symptoms, Physical Discomfort/Sensory Abnormalities, and Stress Relief. Regarding RQ2, the developed scale exhibited an interpretable factor structure and acceptable internal consistency for most subscales, though the Stress Relief subscale’s relatively low reliability and the non-optimal CFA fit indicate the need for further refinement. Regarding RQ3, the MDRES subscales demonstrated theoretically interpretable associations with external variables, including psychological distress, game engagement, absorption, spatial presence, somatic symptoms, and mental health-related indicators.

### 4.2 Theoretical implications

The central contribution of this study is that it highlights the multidimensional and psychosomatic nature of metaverse dependence. Whereas existing measures of problematic technology use have typically focused on gaming, generalized Internet use, smartphones, or social media and emphasized behavioral and cognitive components such as salience, tolerance, withdrawal, and conflict (Young, 1998; Lemmens et al., 2009; Caplan, 2010; Kwon et al., 2013; Kim et al., 2014), the MDRES additionally captures bodily and sleep-related dimensions (e.g., dizziness, cybersickness-like symptoms, musculoskeletal strain, sleep disturbance-like complaints), as well as confusion between physical and virtual spaces and stress-relief experiences characteristic of socially immersive virtual environments. This profile suggests that metaverse-related dependence is not merely a digital extension of conventional gaming or Internet addiction but a more complex pattern that integrates escapism and reality avoidance, immersion-driven boundary blurring, and the embodied costs and benefits of intensive virtual participation.

Simultaneously, the findings may extend behavioral addiction frameworks originally developed with regard to gambling, online gaming, and generalized Internet use (Young, 1998; Griffiths, 2005; Lemmens et al., 2009; Caplan, 2010; Kuss and Griffiths, 2012)–to socially immersive, avatar-based environments. Developed with reference to component models of addiction (Griffiths, 2005; Lemmens et al., 2009), the six-factor structure of the MDRES indicates that metaverse-related dependence is not a unitary construct but consists of partially distinct domains (e.g., reality avoidance, spatial confusion, physical and sleep disturbance-like symptoms). In this sense, “metaverse dependence” is better understood as a constellation of interrelated processes than as a single homogeneous syndrome. Consistent with this view, the term “metaverse dependence” in the present study refers to dependence-like tendencies grounded in behavioral addiction models rather than to a formal *DSM-5* diagnosis; the MDRES is more appropriately conceptualized as capturing a multidimensional profile of risk and adaptation associated with metaverse use than as indexing a categorical disorder.

Moreover, the coexistence of maladaptive and adaptive facets within MDRES has implications for the debate on compensatory and problematic technology use. The joint presence of reality avoidance and stress relief suggests that the same immersive environments may function both as compensatory spaces for dealing with offline stressors and as contexts in which avoidance and overdependence may emerge (Kuss and Griffiths, 2012), supporting a graded view in which high-frequency compensatory use is not automatically equated with “disorder” but may become problematic when combined with sleep disturbance-like symptoms or disorientation. In this sense, the present scale was developed in line with the compensatory use framework (Kardefelt-Winther, 2014) and can be regarded as capturing multiple, partially overlapping dimensions that reflect both functional and dysfunctional aspects of metaverse use.

Finally, by situating metaverse-related dependence at the intersection of technology addiction research and immersion psychology, the MDRES provides a conceptual basis for comparative cross-media research. Used alongside existing Internet and smartphone addiction scales (Young, 1998; Caplan, 2010; Kwon et al., 2013; Kim et al., 2014), it enables examination of whether metaverse dependence represents a distinct subtype, a severity marker within a broader spectrum of technology-related problems, or a cross-cutting vulnerability pattern characterized by features such as high embodiment, time distortion, and continuity of identity across virtual and physical realities (Barreda-Ángeles and Hartmann, 2022; Dincelli and Yayla, 2022).

### 4.3 Practical implications

At this stage, the MDRES should be regarded as a research instrument rather than a clinical screening or decision-making tool. As metaverse platforms become embedded not only in entertainment but also in education and business (PWC, 2022), and as their use expands, particularly among younger individuals (Demand Sage, 2025), projections that a substantial proportion of the global population will spend time in the metaverse daily (Gartner, 2022; Grand View Research Inc, 2024; Coolest Gadgets, 2025) further underscore the need for tools that provide feasible, multidimensional assessments of psychosomatic symptoms and dependence-like tendencies. Nevertheless, the present findings may provide a preliminary basis for the future refinement and validation of the MDRES as a measure of metaverse-related risk profiles. Longitudinal and clinically anchored validation will be necessary before the MDRES can be considered for screening or clinical decision-making.

At the individual functioning level, the present findings suggest the possible coexistence of potentially adaptive and maladaptive experiences associated with metaverse use. The coexistence of reality avoidance and stress relief within the factor structure, alongside their distinct external correlates, suggests that the same social VR environments can simultaneously serve as spaces for stress reduction and contexts in which avoidance, sleep disruption, and psychosomatic strain may be reported. This pattern aligns with the compensatory-use perspective, which proposes that individuals may turn to online environments to regulate mood and manage offline stressors; however, such coping can become maladaptive under certain conditions (Kuss and Griffiths, 2012; Kardefelt-Winther, 2014).

### 4.4 Limitations and future directions

Because the present study employed a correlational cross-sectional design, causal inference was inherently limited. Accordingly, longitudinal and experimental approaches are required to probe temporal dynamics, thresholds, and mechanisms (e.g., whether sleep disturbance precedes or follows over immersion). Regarding sampling, participants were predominantly young and self-selected metaverse users, constraining generalizability across age groups, cultural contexts, device modalities (head-mounted display versus desktop/mobile), and platform types (social versus game-centric). Although a sizable proportion of participants reported using head-mounted displays such as PC-connected or standalone VR goggles (see Supplementary Table 7), the data were not designed to test modality-specific measurement invariance.

On the measurement side, the initially specified six-factor CFA model did not demonstrate adequate overall fit. A modified model showed substantially improved and generally adequate fit after 32 cross-loadings were sequentially freed based on the modification indices. However, because their selection and estimation were conducted using the same CFA sample, the modified model should be regarded as data-informed and exploratory rather than as an independently confirmed model. Future studies should refine the item pool and examine whether the modified factor structure, including the cross-loadings retained in the modified model, can be replicated in a new independent sample. Thus, item refinement and expansion of the item pool are warranted to enhance reliability and content coverage. In particular, future studies should revise and expand the item pool for the Stress Relief subscale (Supplementary Indicator) and re-evaluate its reliability and construct validity in independent samples. Additionally, future item development should directly assess core dependence features, including impaired control, tolerance, withdrawal, salience, and persistence despite harm, to determine whether the MDRES can be extended to assess clinical dependence more comprehensively.

Considering the intercorrelations among the subscales, more stringent tests of discriminant validity–comparisons with theoretically related measures–are recommended, ideally using independent and larger samples. As the extracted factors reflected heterogeneous domains, including behaviors, thoughts, physical reactions, and perceived consequences, future studies should examine whether these domains constitute a stable multidimensional construct. This research did not directly examine the relationship between the MDRES and established measures of GTP, such as the Game Transfer Phenomena Scale or the GTP-MDS. Moreover, GTP research has primarily focused on the involuntary transfer of gaming experiences into daily life, whereas the MDRES assesses a broader range of metaverse dependency effects as they appear within real-world contexts. Future studies should assess associations between the MDRES and GTP-related measures to determine whether metaverse-related confusion between physical and virtual spaces represents a distinct phenomenon or an extension of previously identified GTP experiences.

To mitigate the common-method bias inherent in self-reporting measures, it is advisable to integrate objective usage logs (e.g., session duration, latency, and locomotion settings), physiological indices (e.g., heart-rate variability), and behavioral indices (e.g., eye tracking). To address the risk of false-positive findings arising from multiple correlation tests, the BH false discovery rate correction was applied separately to each predefined family of analyses. Nevertheless, because the associations with external variables and the SSS-8 item-level analyses were exploratory, these findings require replication in independent samples.

Furthermore, future work should establish measurement invariance across languages and cultures using rigorous translation/back-translation procedures, demonstrate predictive validity (e.g., prospective risk for functional impairment), consider the development of cut scores where appropriate, and evaluate intervention sensitivity (e.g., pre–post changes following design modifications or clinical programs). Currently, the MDRES is best regarded as a preliminary research instrument that captures a multidimensional profile of metaverse-related dependency risk, associated psychosomatic experiences, and perceived benefits, rather than as a diagnostic tool with established clinical cutoffs.

## 5 Conclusion

In this study, we developed the MDRES, examined its six-factor structure through a pilot study and two questionnaire surveys, and provided preliminary evidence for its reliability and validity. The MDRES was developed not as a screening tool for metaverse dependence, but as a preliminary multidimensional scale to measure metaverse-related dependency risks and associated physical and psychological symptoms that manifest in real-world contexts. It comprises the following six subscales: Reality Avoidance, Confusion Between Real and Virtual Spaces, Awareness of Physical Symptoms, Sleep Disturbance-Like Symptoms, Physical Discomfort/Sensory Abnormalities, and Stress Relief (Supplementary Indicator). By extending behavioral addiction frameworks to the metaverse context, the MDRES conceptualizes metaverse-related dependence as a multifaceted phenomenon that encompasses not only emotional and cognitive processes but also psychosomatic processes.

## Data Availability

The datasets presented in this article are not readily available because legal and ethical restrictions imposed by “Development of Foundational Technologies and Rules for Expansion of the Virtual Economy” funded by New Energy and Industrial Technology Development Organization (NEDO), the data supporting this study is not allowed to be shared publicly. Requests for data access may be considered on a case-by-case basis. Requests to access the datasets should be directed to TH, homma.takuto@aist.go.jp.
