# Supplementary material for: Metaverse dependency as a psychosomatic risk of immersive platforms: development and validation of the Metaverse Dependency-Related Experiences Scale
Source: Front Psychol. 2026 Sep 23;17:1814271. doi: 10.3389/fpsyg.2026.1814271 (PMC13642638; doi:10.3389/fpsyg.2026.1814271)
Supplement: Supplementary file 1 [file Data_Sheet_1.pdf]

Supplementary Material

1 Supplementary Tables

Supplementary Table 1. Free-text responses in the pilot study

No.	Items
1	What do you usually do in the metaverse space? Please write as specifically as possible. Please answer as extensively as possible.
2	Compared with real spaces, are there any behaviors or events that are unique to metaverse spaces? Please write freely and as specifically as possible (e.g., “sleeping in VR”). Please list as many as you can think of.
3	Have you ever ended up doing something in real space that you usually do in a metaverse space? What specifically did you do (e.g., “placing an object in midair”)? Please list as many as you can think of.
4	Regarding communication in metaverse spaces, what advantages do you think it has compared with communication in real spaces? Please list as many as you can think of.
5	Regarding communication in metaverse spaces, what disadvantages do you think it has compared with communication in real spaces? Please list as many as you can think of.
6	Are there any differences in the way you communicate in metaverse versus real spaces? Please write freely and as specifically as possible. Please list as many as you can think of.
7	Did spending time in metaverse spaces change your real-life relationships, such as relationships with family or friends? Please list as many changes as you can think of.
8	When spending time in metaverse spaces, have you experienced any physical discomfort that appears only in metaverse versus real spaces (e.g., headaches or eye fatigue)? Please list as many as you can think of.
9	When spending time in metaverse spaces, have you experienced any psychological discomfort that appears only in metaverse spaces compared with real spaces (e.g., feeling depressed or becoming irritated)? Please list as many as you can think of.

10	When spending time in metaverse spaces, have there been any positive changes in your lifestyle habits (e.g., improved eating habits or sleeping better)? Please list as many as you can think of.
11	When spending time in metaverse spaces, have there been any negative changes in your lifestyle habits (e.g., disrupted eating habits or shorter sleep time)? Please list as many as you can think of.
12	When spending time in metaverse spaces, have there been any changes in your values or way of thinking? Please list as many as you can think of.
13	Why do you want to prioritize [metaverse or real-world space]? Please list as many reasons as you can think of.
14	What do you usually do in the metaverse space? Please write as specifically as possible. Please answer as extensively as possible.
15	Compared with real spaces, are there any behaviors or events that are unique to metaverse spaces? Please write freely and as specifically as possible (e.g., “sleeping in VR”). Please list as many as you can think of.

Supplementary Table 2. Full item wordings for the Metaverse Dependency-Related Experiences Scale

No.	Items
1	I have come to feel eye strain.
2	I have come to have stiff shoulders.
3	I stay up late more often.
4	I have come to have a stiff neck.
5	My sleep time has decreased.
6	My stress has decreased.
7	I get insufficient exercise.
8	I have become sleep-deprived.
9	I have come to feel that my head gets tired.
10	I have come to feel that I am slouching.
11	My eyesight has declined.
12	I have developed dry eyes.
13	I have come to feel fatigued.
14	I use virtual space to escape from reality.
15	I have come to feel a decline in physical stamina.
16	I have come to feel an elevated mood more often.
17	I have come to feel that I am losing my sense of time.
18	I have come to feel sluggish in my body.

19	Socializing has come to feel bothersome.
20	The time I spend talking with friends has decreased.
21	I have come to have blurred vision.
22	My daily habits have become disordered.
23	The time I spend talking with family has decreased.
24	I have come to feel that I have a place only in virtual space.
25	I end up using words that I often use in virtual space in real space as well.
26	Everything has come to feel bothersome.
27	I have become poor at communication in real space.
28	I end up interacting in real space with the same psychological sense of distance as in virtual space.
29	My interpersonal relationships have become more tenuous.
30	I have become impatient.
31	I end up communicating in the same way in real space as in virtual space.
32	My eating habits have become disordered.
33	I have come to feel motion sickness.
34	I have come to have headaches.
35	I have come to have ear pain.
36	I become irritated more often.
37	My sleep quality has improved.
38	I have come to feel lightheaded when standing up.

39	I have come to feel depressed more often.
40	I end up keeping the same physical sense of distance in real space as in virtual space.
41	I end up making the same movements for moving around in real space as I do in virtual space.
42	I end up hearing music or sounds from virtual space in real space as well.
43	My cautiousness has been lost.
44	I end up making the same gestures in real space as in virtual space.
45	I end up making the same facial expression movements in real space as in virtual space.
46	I have come to feel loneliness.
47	I end up recognizing the laws of physics in real space in the same way as in virtual space.
48	I become anxious when I am not in virtual space.
49	I have come to feel that my bodily movements in real space have become clumsy.
50	I have come to feel down more often.
51	I find myself about to touch things in real space that should not be touched.
52	It has become difficult to control my emotions.

Supplementary Table 3. Frequencies and means (*SD*) of sociodemographic information

Please tell us your age.	Age	37.40 (8.38)
Please tell us your sex.	Male	213
	Female	112
Are you married?	Married	183
	Unmarried	142
Please tell us your occupation.	Part-time worker, temporary worker, non-regular employee	32
	Company employee/executive	179
	Civil servant	2
	Home-based piecework	1
	Student	2
	Stay-at-home parent	11
	Professional/specialist occupation (e.g., physician/doctor, lawyer/attorney, hairstylist/hairdresser, designer)	6
	Unemployed (including retirees)	5
	Self-employed/free enterprise	87
Please list the name of the metaverse content you use most frequently.	VRChat	202
	ZEPETO	43

	cluster	39
	Second Life	14
	Rec Room	9
	Bondee	6
	Avakin Life	1
	DOOR	1
	IMVU	1
	Memotia (Memoria)	1
	Microsoft Mesh	1
	REALITY	1
	VRChat, cluster	1
	XR CLOUD	1
	Virtual Matsumoto	1
	HOLOEARTH	1
	Metaverse office	1
	Hiroshima Toyo Carp “Meta-Carp”	1
Please list any other metaverse content or services you use.	No other content	298
	cluster	5
	VRChat	4
	Fortnite	4

	ZEPETO	2
	Bondee	1
	Minecraft	1
	REALITY	1
	Roblox	1
	Roblox, Metaverse	1
	SHIB: The Metaverse	1
	cluster, Fortnite	1
	Animal Crossing: New Horizons	1
	The Sims	1
	Parallel	1
	Fortnite, Minecraft	1
Please indicate how long you have been using the metaverse content you use most frequently.	Length of use (years)	1.69 (*SD* = 1.45)

Supplementary Table 4. Frequency of prefecture

Please tell us your address.	Frequency
Hokkaido	22
Aomori	0
Iwate	5
Miyagi	6
Akita	2
Yamagata	5
Fukushima	3
Ibaraki	7
Tochigi	1
Gunma	3
Saitama	19
Chiba	13
Tokyo	53
Kanagawa	28
Niigata	6
Toyama	7
Ishikawa	7
Fukui	2
Yamanashi	5

Nagano	5
Gifu	5
Shizuoka	6
Aichi	10
Mie	4
Shiga	7
Kyoto	4
Osaka	20
Hyogo	17
Nara	3
Wakayama	3
Tottori	3
Shimane	2
Okayama	7
Hiroshima	6
Yamaguchi	2
Tokushima	0
Kagawa	2
Ehime	2
Kochi	0

Fukuoka	13
Saga	1
Nagasaki	0
Kumamoto	1
Oita	1
Miyazaki	1
Kagoshima	3
Okinawa	2
Other	1

Supplementary Table 5. Item-level descriptive statistics for the Metaverse Dependency-Related Experiences Scale

No.	Items	Mean	*SD*	Min.	Max.
1	I have come to feel eye strain.	3.42	1.09	1.00	5.00
2	I have come to have stiff shoulders.	3.25	1.14	1.00	5.00
3	I stay up late more often.	3.17	1.21	1.00	5.00
4	I have come to have a stiff neck.	3.16	1.20	1.00	5.00
5	My sleep time has decreased.	3.13	1.18	1.00	5.00
6	My stress has decreased.	3.12	0.96	1.00	5.00
7	I get insufficient exercise.	3.02	1.13	1.00	5.00
8	I have become sleep-deprived.	3.01	1.25	1.00	5.00
9	I have come to feel that my head gets tired.	2.90	1.13	1.00	5.00
10	I have come to feel that I am slouching.	2.88	1.23	1.00	5.00
11	My eyesight has declined.	2.86	1.13	1.00	5.00
12	I have developed dry eyes.	2.84	1.13	1.00	5.00
13	I have come to feel fatigued.	2.84	1.08	1.00	5.00
14	I use virtual space to escape from reality.	2.73	1.20	1.00	5.00
15	I have come to feel a decline in physical stamina.	2.68	1.12	1.00	5.00
16	I have come to feel an elevated mood more often.	2.65	1.00	1.00	5.00
17	I have come to feel that I am losing my sense of time.	2.62	1.15	1.00	5.00
18	I have come to feel sluggish in my body.	2.61	1.13	1.00	5.00

19	Socializing has come to feel bothersome.	2.60	1.15	1.00	5.00
20	The time I spend talking with friends has decreased.	2.55	1.16	1.00	5.00
21	I have come to have blurred vision.	2.54	1.18	1.00	5.00
22	My daily habits have become disordered.	2.51	1.09	1.00	5.00
23	The time I spend talking with family has decreased.	2.51	1.13	1.00	5.00
24	I have come to feel that I have a place only in virtual space.	2.44	1.21	1.00	5.00
25	I end up using words that I often use in virtual space in real space as well.	2.43	1.07	1.00	5.00
26	Everything has come to feel bothersome.	2.43	1.14	1.00	5.00
27	I have become poor at communication in real space.	2.40	1.16	1.00	5.00
28	I end up interacting in real space with the same psychological sense of distance as in virtual space.	2.39	0.98	1.00	5.00
29	My interpersonal relationships have become more tenuous.	2.38	1.10	1.00	5.00
30	I have become impatient.	2.36	1.09	1.00	5.00
31	I end up taking the same way of communicating in real space as in virtual space.	2.33	1.01	1.00	5.00
32	My eating habits have become disordered.	2.33	1.14	1.00	5.00
33	I have come to feel motion sickness.	2.33	1.14	1.00	5.00
34	I have come to have headaches.	2.32	1.15	1.00	5.00
35	I have come to have ear pain.	2.31	1.19	1.00	5.00
36	I become irritated more often.	2.28	1.04	1.00	5.00

37	My sleep quality has improved.	2.27	0.93	1.00	5.00
38	I have come to feel lightheaded when standing up.	2.27	1.19	1.00	4.00
39	I have come to feel depressed more often.	2.26	1.13	1.00	5.00
40	I end up keeping the same physical sense of distance in real space as in virtual space.	2.23	1.07	1.00	5.00
41	I end up making the same movements for moving around in real space as I do in virtual space.	2.23	1.11	1.00	5.00
42	I end up hearing music or sounds from virtual space in real space as well.	2.21	1.10	1.00	5.00
43	My cautiousness has been lost.	2.19	1.02	1.00	5.00
44	I end up making the same gestures in real space as in virtual space.	2.19	1.01	1.00	5.00
45	I end up making the same facial expression movements in real space as in virtual space.	2.17	1.03	1.00	5.00
46	I have come to feel loneliness.	2.16	0.98	1.00	5.00
47	I end up recognizing the laws of physics in real space in the same way as in virtual space.	2.16	1.00	1.00	5.00
48	I become anxious when I am not in virtual space.	2.14	1.03	1.00	5.00
49	I have come to feel that my bodily movements in real space have become clumsy.	2.14	1.07	1.00	5.00
50	I have come to feel down more often.	2.12	0.91	1.00	5.00
51	I find myself about to touch things in real space that should not be touched.	2.02	1.02	1.00	5.00
52	It has become difficult to control my emotions.	1.99	1.01	1.00	5.00

Supplementary Table 6. Heterotrait–monotrait ratios of correlations among the six Metaverse Dependency-Related Experiences Scale factors

Factor intercorrelations	F1	F2	F3	F4	F5	F6
F1	—	0.74	0.47	0.77	0.43	0.40
F2		—	0.36	0.68	0.46	0.44
F3			—	0.58	0.68	0.47
F4				—	0.47	0.37
F5					—	0.37
F6						—

Note. F1 = Reality Avoidance; F2 = Confusion Between Physical and Virtual Spaces; F3 = Awareness of Physical Symptoms; F4 = Sleep Disturbance-Like Symptoms; F5 = Physical Discomfort/Sensory Abnormalities; F6 = Stress Relief. Values below .85 were considered as indicative of discriminant validity (Henseler et al., 2015).

Supplementary Table 7. Frequency and mean (*SD*) of sociodemographic information

Please tell us your age.	Age	36.58 (10.16)
Please tell us your sex.	Male	123
	Female	68
Are you married?	Married	80
	Unmarried	111
Please tell us your occupation.	Company employee/executive	114
	Part-time worker, temporary worker, non-regular employee	22
	Self-employed/free enterprise	14
	Student	13
	Unemployed (including retirees)	10
	Civil servant	7
	Stay-at-home parent	7
	Professional/specialist occupation (e.g., physician/doctor, lawyer/attorney, hairstylist/hairdresser, designer)	4
	Other	0
Please list the name of the metaverse content you use most frequently.	VRChat	140
	ZEPETO	25

	cluster	13
	Bondee	3
	Rec room	2
	Second Life	4
	REVWORLD	1
	VRChat, ZEPETO, Bondee	1
	HOLOEARTH	1
	ZEPETO, Parallel, cluster	1
Please list any other metaverse content or services you use.	No other content	176
	VRChat	2
	Fortnite	2
	ZEPETO	2
	Bondee	1
	cluster	1
	ZEPETO	1
	Parallel	1
	Gemini	1
	Chat GPT	1
	asi	1
	YouTube	1

	Forgotten	1
Which would you prefer: the metaverse or the real world?	Metaverse space	92
	Real-world space	99
What devices do you typically use when accessing metaverse content? Please select the option that best applies to you.	PC・Desktop	59
	VR goggles (PC connection available)	73
	VR goggles (No PC connection)	11
	Smartphone	32
	Game console	13
	Smart glasses	3
How many hours per day, on average, do you spend using metaverse content?	Average daily usage time	1.74 (1.76)
Please indicate how long you have been using the metaverse content you use most frequently.	Length of use (years)	2.06 (1.87)

Supplementary Table 8. Item-level standardized factor loadings of confirmatory factor analysis

Factor	Items (short)	Standardized factor loadings
F1	Everything feels bothersome	0.83
	Weaker relationships	0.81
	Poor real-space communication	0.83
	Less time with friends	0.80
	Socializing feels bothersome	0.62
	Depressed more often	0.82
	Feeling down more often	0.71
	Feeling lonely	0.75
	Irritated more often	0.74
	Less time with family	0.72
	More impatient	0.63
F2	Almost touch off-limits objects	0.69
	Same interpersonal distance (real–virtual)	0.79
	Virtual physics applied to real	0.77
	Same locomotion (real–virtual)	0.72
	Virtual sounds heard in real	0.60
	Same facial expressions (real–virtual)	0.77
	Same communication style (real–virtual)	0.80
	Use virtual terms in real	0.69

	Same gestures (real–virtual)	0.75
F3	Stiff shoulders	0.73
	Eye strain	0.66
	Stiff neck	0.67
	Head fatigue	0.66
	Slouching posture	0.61
	Decline in stamina	0.73
	Worsened eyesight	0.63
F4	Cybersickness	0.60
	Ear pain	0.73
	Headaches	0.78
	Physical sluggishness	0.72
	Dizzy on standing	0.79
F5	Disordered daily routines	0.63
	Sleep-deprived	0.86
	Staying up late	0.84
	Less sleep time	0.84
F6	Less stress	0.42
	Better sleep quality	0.34
	Elevated mood more often	0.80

Supplementary Table 9. Factor intercorrelations of confirmatory factor analysis

	F1	F2	F3	F4	F5	F6
F1	—	0.73	0.44	0.76	0.34	0.33
F2		—	0.34	0.69	0.40	0.46
F3			—	0.55	0.66	0.31
F4				—	0.42	0.16
F5					—	0.38
F6						—

Supplementary Table 10. Item-level standardized factor loadings of confirmatory factor analysis of modified measurement model

Factor	Items (short)	Standardized factor loadings
	Everything feels bothersome	0.83
	Weaker relationships	0.81
	Poor real-space communication	0.85
	Less time with friends	0.81
	Socializing feels bothersome	0.92
F1	Depressed more often	0.83
	Feeling down more often	0.72
	Feeling lonely	0.75
	Irritated more often	0.73
	Less time with family	0.74
	More impatient	0.61
	Almost touch off-limits objects	0.69
	Same interpersonal distance (real–virtual)	0.57
	Virtual physics applied to real	0.57
F2	Same locomotion (real–virtual)	0.72
	Virtual sounds heard in real	0.61
	Same facial expressions (real–virtual)	0.77
	Same communication style (real–virtual)	0.80

	Use virtual terms in real	0.70
	Same gestures (real–virtual)	0.92
	Stiff shoulders	0.73
	Eye strain	0.74
	Stiff neck	0.62
F3	Head fatigue	0.43
	Slouching posture	0.45
	Decline in stamina	0.63
	Worsened eyesight	0.56
	Cybersickness	0.59
	Ear pain	0.74
F4	Headaches	0.78
	Physical sluggishness	0.67
	Dizzy on standing	0.93
	Disordered daily routines	0.49
	Sleep-deprived	0.87
F5	Staying up late	0.84
	Less sleep time	0.84
F6	Less stress	0.70
	Better sleep quality	0.52

	Elevated mood more often	0.43
	Additional Standardized Cross-Loadings	
F1	Disordered daily routines	0.36
	Slouching posture	0.43
	Decline in stamina	0.42
	Worsened eyesight	0.28
	Virtual physics applied to real	0.32
	Same interpersonal distance (real–virtual)	0.29
	Stiff shoulders	0.15
	Same gestures (real–virtual)	-0.20
F2	Almost touch off-limits objects	0.36
F3	Physical sluggishness	0.28
	Socializing feels bothersome	0.43
	Dizzy on standing	-0.33
	More impatient	0.22
	Weaker relationships	0.15
	Irritated more often	0.11
	Elevated mood more often	0.13
F4	Head fatigue	0.49
	Stiff neck	0.23

	Socializing feels bothersome	-0.42
	Better sleep quality	0.48
F5	Better sleep quality	-0.33
	Virtual physics applied to real	-0.11
F6	Less time with family	0.30
	Less time with friends	0.23
	Same communication style (real–virtual)	0.13
	Poor real-space communication	0.16
	Weaker relationships	0.16
	Dizzy on standing	0.14
	Disordered daily routines	0.15
	Irritated more often	-0.21
	Depressed more often	-0.13
	Head fatigue	-0.14

Supplementary Table 11. Factor intercorrelations of confirmatory factor analysis of modified measurement model

	F1	F2	F3	F4	F5	F6
F1	—	0.69	0.00	0.75	0.25	- 0.10
F2		—	0.05	0.66	0.39	0.02
F3			—	0.23	0.61	0.13
F4				—	0.39	- 0.29
F5					—	0.18
F6						—
